# Supplementary material for: Recent reactivation of a pathogenicity-associated transposable element is associated with major chromosomal rearrangements in a fungal wheat pathogen
Source: Nucleic Acids Res. 2023 Dec 24;52(3):1226–42. doi: 10.1093/nar/gkad1214 (PMC10853768; doi:10.1093/nar/gkad1214)
Supplement: gkad1214_Supplemental_Files [file gkad1214_supplemental_files.zip › Supplementary_Data.pdf]

Supplementary Data

Data S1: Sequences of the Terminal Inverted Repeats used for transposon re-annotation using *packfinder*.

>DXX_Styx
ACGGACGACTGGTAGAACAATAGCTGCAAGAACTCG

>DTA_Vera
CAGTCTGCTAAACAATCAAT

>DTT_Tapputi
AGAAGTCACTTGCAAGGTATGCACGGTGCACGCTGTGCACGGTCGCA

>DXX_Birute
ACTCGTGTTGCAAGCTTCTCACCGCCAAAGCGGTGTTGGCCGGCTCAATGAGAATCACCA
GCTCAACACGACTGTATC
