# Supplementary material for: Recent reactivation of a pathogenicity-associated transposable element is associated with major chromosomal rearrangements in a fungal wheat pathogen
Source: Nucleic Acids Res. 2023 Dec 24;52(3):1226–42. doi: 10.1093/nar/gkad1214 (PMC10853768; doi:10.1093/nar/gkad1214)
Supplement: gkad1214_Supplemental_Files [file gkad1214_supplemental_files.zip › Supplementary_Figures.pdf]

Supplementary Figures

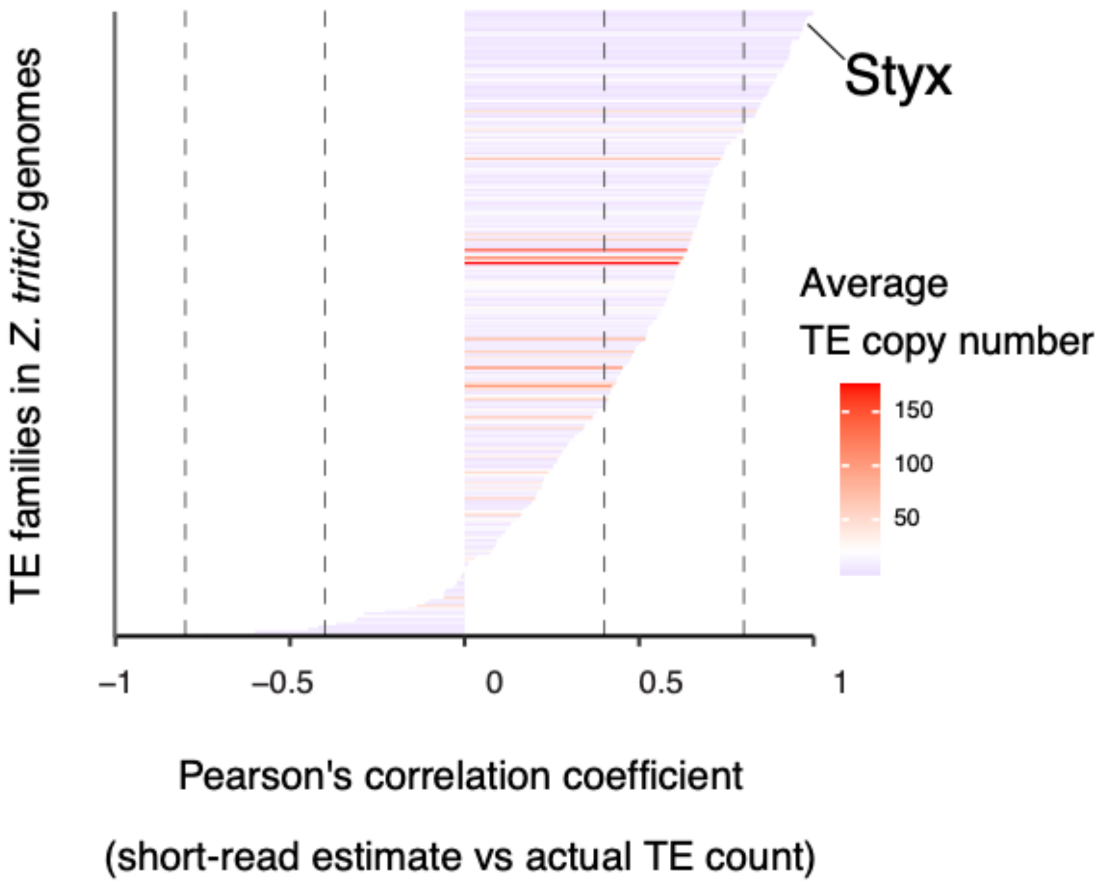

Figure S1: Correlation between the estimated copy number from short reads and long reads assemblies of ten *Zymoseptoria tritici* strains. Bars represent the correlation for each of the individual transposable element families identified in the analyzed genomes. The *Styx* element is highlighted and shows among the highest correlations between actual TE count and short-read estimate.

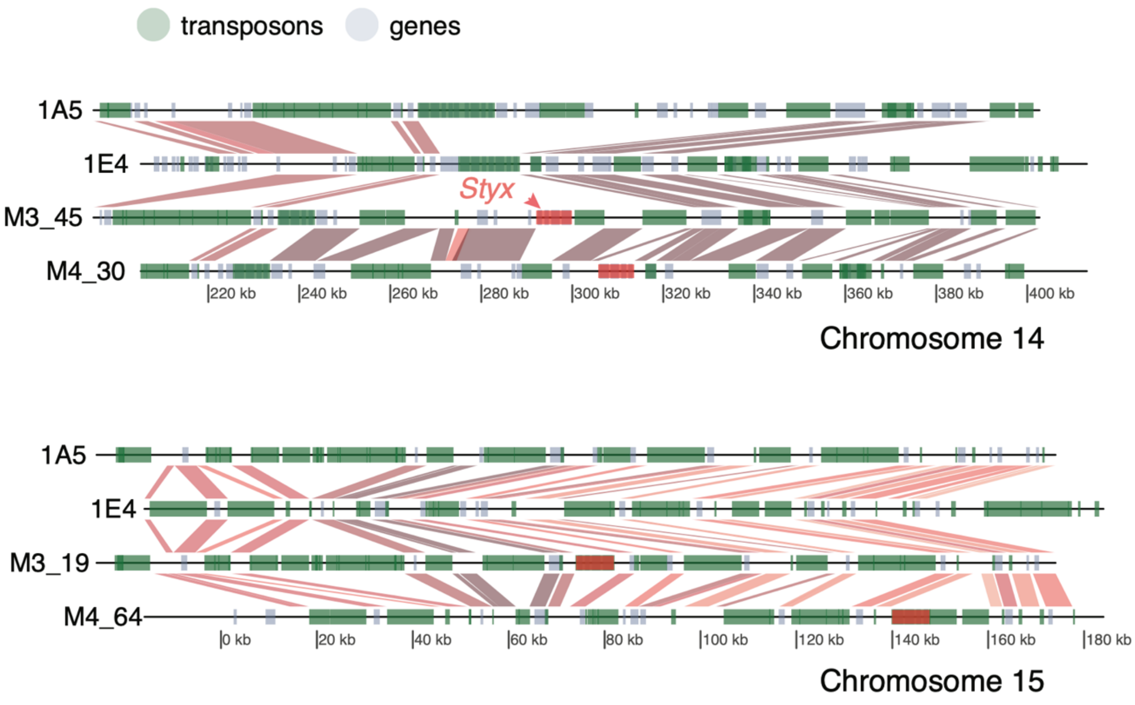

Figure S2: Synteny plots illustrating recurrent *de novo Styx* insertions in close proximity in the *Zymoseptoria tritici* pedigree. Isolates 1A5 and 1E4 are the parents and M3_45, M3_19, M4_30 and M4_64 are the progeny from the third (M3) and fourth (M4) round of meiosis. *Styx* elements, TEs and genes are highlighted by coloured boxes.

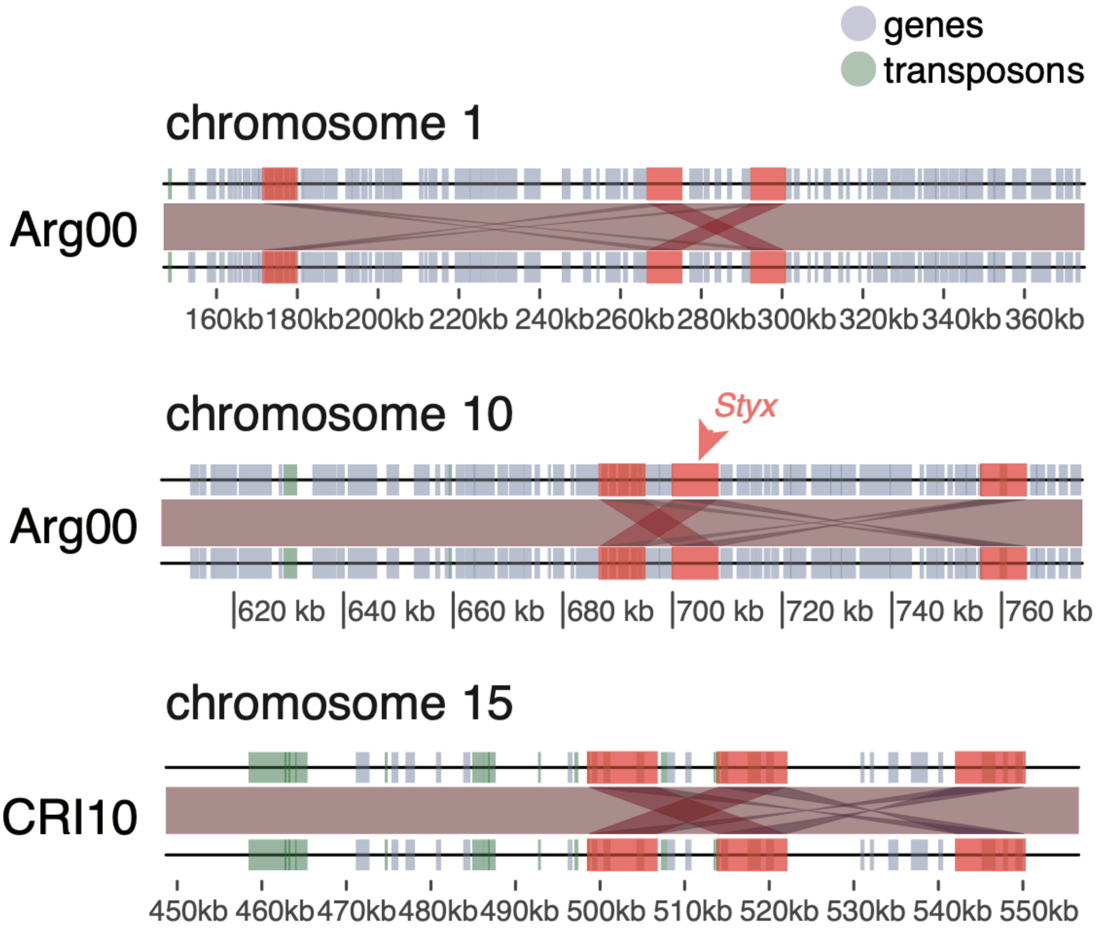

Figure S3: Illustration of the recurrent pattern of *Styx* tandem insertions along a same chromosome. Plots show the synteny for the same chromosome and isolate. *Styx* elements, TEs and genes are highlighted by coloured boxes.

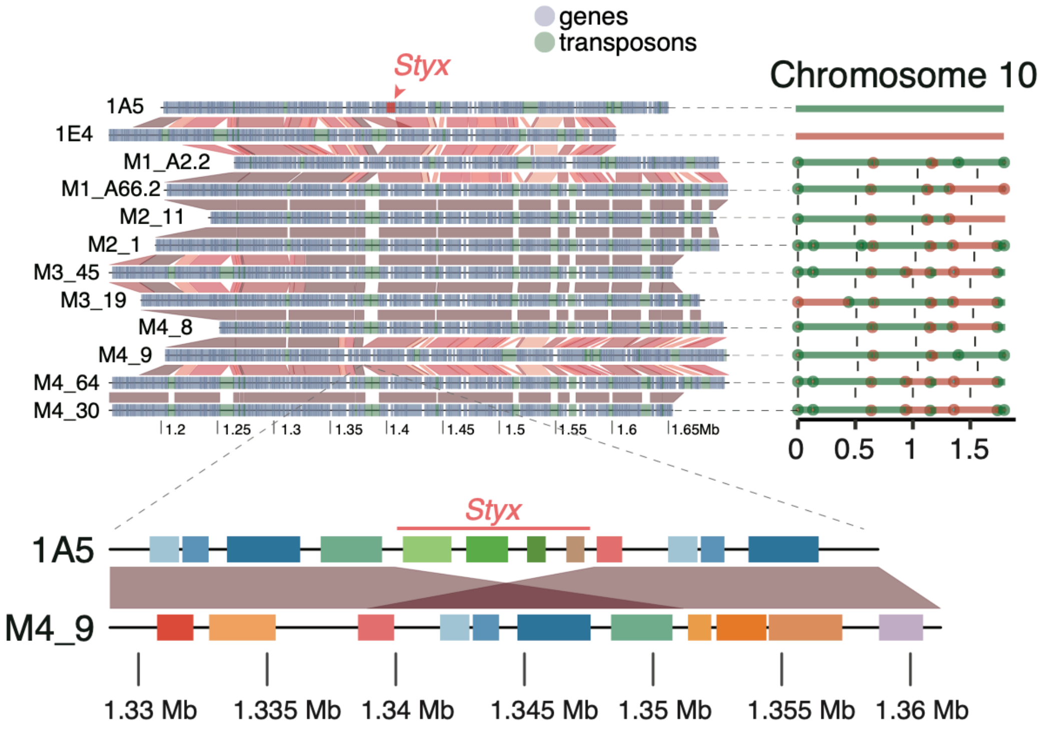

Figure S4: Independent excision events of a *Styx* copy on chromosome 10 in the pedigree. Plots show the synteny for the end of chromosome 10 in all progeny isolates. The *Styx* copy present in the 1A5 parent is highlighted in red. Dot-lines on the right side illustrate the recombination events that took place during the crosses. Colours indicate the parental origin of the chromosome segment. The lower synteny plot is a zoom in the excision event in progeny isolate M4_9. The four *Styx* coding sequences are highlighted, and the three surrounding duplicated genes are shown in same coloured shades.
